# Using a Multi-Tracer Approach to Examine Perfluoroalkyl Substance Sources and Dietary Exposure Pathways in Pacific Bald Eagles

**DOI:** 10.1021/acs.est.5c15624

**Published:** 2026-03-17

**Authors:** Robert Kesic, John E. Elliott, Myles Lamont, Sandi L. Lee, Kimberly M. Cheng, France Maisonneuve

**Affiliations:** † 6347 Environment and Climate Change Canada, Wildlife Research Division, Delta, British Columbia V4K 3N2, Canada; ‡ TerraFauna Wildlife Consulting; Hancock Wildlife Foundation, Surrey, British Columbia V3Z 9R9, Canada; § 8166 University of British Columbia, Applied Animal Biology, Vancouver, British Columbia V6T 1Z4, Canada; ∥ Environment and Climate Change Canada, National Wildlife Research Centre (NWRC), Ottawa, Ontario K1S 5R2, Canada

**Keywords:** bald eagles, perfluoroalkyl substances, PFAS, PFOS, PFCAs, stable isotopes, fatty acids, bioaccumulation

## Abstract

Perfluoroalkyl substances (PFAS) are globally distributed contaminants that bioaccumulate in avian apex predators, yet species-specific bioaccumulation processes and links to dietary sources remain poorly resolved. Using an integrative, multi-habitat sampling framework, we blood sampled 89 bald eagle (*Haliaeetus leucocephalus*) nestlings across eight regions from the Pacific coast of British Columbia (BC), Canada and quantified 17 PFAS. We paired contaminant data with stable isotope analyses (δ^15^N, δ^13^C, δ^34^S) to characterize trophic position and habitat use, and fatty acid (FA) profiling to partition aquatic vs terrestrial prey sources, while modeling biological covariates. Our most parsimonious model describing spatial variation in PFAS exposure included region and nestling age. PFAS profiles were dominated by PFOS (mean = 23.5; range 2.1–159 ng/mL), comprising 33–72% of ∑_17_ PFAS, with remaining burdens composed of PFNA, PFDA, PFUdA, and PFTrDA. Stable isotopes did not uniformly predict PFAS concentrations; however, PFUdA and PFTrDA were positively correlated with δ^13^C and ∑ Omega-3 FAs, consistent with marine-derived diets. PFOS and PFHxS were negatively correlated with ∑ Omega-6 FAs, suggesting greater biomagnification in nestlings feeding within terrestrial or mammalian food webs. To our knowledge, this work represents the first comprehensive assessment of PFAS exposure in Pacific bald eagles and is also one of the first attempts to apply a multi-dietary tracer approach to contextualize habitat use and dietary pathways of PFAS in a regional apex predator.

## Graphical abstract

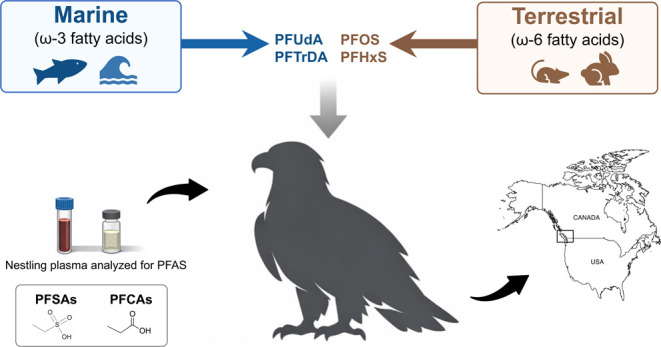

## Introduction

Perfluoroalkyl substances (PFAS), including perfluoroalkyl sulfonates (PFSAs) and perfluoroalkyl carboxylates (PFCAs), are a class of synthetic fluorinated compounds whose carbon–fluorine bonds impart exceptional environmental persistence and which generally have a strong tendency to bioaccumulate and biomagnify through aquatic and terrestrial food webs.^1–4^ Although global regulatory restrictions have reduced legacy PFAS (e.g., perfluorooctanesulfonic acid; PFOS) emissions and exposure in some wildlife,^5–8^ temporal trends for other PFAS, including long-chain PFCAs, have not been universally consistent, both near source areas^9^ and in remote regions.^10,11^ Accordingly, spatial and temporal trends of PFAS concentrations in wildlife are influenced in part by differences in carbon chain length, diet, habitat use, and tissue-specific partitioning;^12–14^ however, relative to lower-trophic level species, the mechanisms governing PFAS exposure remain poorly resolved in avian apex predators, where high trophic positions, broad feeding ranges, and long life spans may promote greater biomagnification potential.^1,2,14^

Avian apex predators are important sentinels for assessing the bioaccumulation and trophic transfer of PFAS, yet our understanding of PFAS in such species remains, in part, geographically and taxonomically biased. Efforts to characterize PFAS in avian predators have largely been confined to the Atlantic,^8,15^ Arctic,^7,16^ Great Lakes,^17–19^ Mediterranean,^20^ and select Eurasian regions.^9,10,21^ By contrast, PFAS data from western North American avifauna remain scarce, despite the region’s industrialized coastlines and susceptibility to long-range transport (LRT) of contaminants from local and global sources.^4,5,22,23^ The Salish Sea, encompassing Puget Sound, the Strait of Georgia, and adjacent coastal waters of Washington State and British Columbia (BC), is bordered by a rapidly urbanizing population exceeding nine million people and is subject to urban runoff, effluent discharge, and dense marine traffic, which are known sources and pathways of PFAS,^5,24^ yet has received comparatively little attention with respect to PFAS. Although PFAS concentrations have declined in some species from the northeast Pacific coast,^5,25^ recent evidence suggests that concentrations of long-chain PFCAs, such as perfluorononanoic acid (PFNA), perfluoroundecanoic acid (PFUdA), and perfluorotridecanoic acid (PFTrDA), are either plateauing or increasing in coastal and freshwater birds from the Salish Sea, including double-crested cormorants (*Nannopterum auritum*)^5^ and glaucous-winged gulls (*Larus glaucescens*).^26^ Whether PFAS patterns in birds from the Salish Sea, and more broadly the Pacific Ocean, represent differences in atmospheric versus oceanic sources, diet, and/or bioaccumulation patterns therefore remains unclear. To address these knowledge gaps and better understand PFAS exposure in anthropogenically impacted coastal ecosystems, we investigated PFAS exposure using the bald eagle (BAEA; *Haliaeetus leucocephalus*) as an apex avian sentinel. Bald eagles are long-lived, feed at high trophic levels, and are broadly distributed throughout their annual cycle.^27–29^ These traits, along with their generalist diet, year-round residency, and site fidelity, make them highly suited for integrating contaminant exposure, both spatially and temporally.^30,31^ Additionally, because adult bald eagles maintain small territories during the breeding season (∼2 km^2^),^32^ nestlings can be used as nondestructive sampling media for assessing local exposure. Nestling sampling also facilitates large sample sizes due to dense breeding populations, as well as high statistical power, enabling reliable detections of spatial patterns, environmentally relevant concentrations, and potential effects.^30,33–36^

To contextualize the dietary pathways of PFAS to bald eagles, we used multiple dietary tracers. Stable isotopes of carbon (δ^13^C) and nitrogen (δ^15^N) were used to infer habitat use and relative trophic position, respectively.^30,37^ Because δ^13^C values can overlap across freshwater and terrestrial systems, we also included stable isotopes of sulfur (δ^34^S) as a secondary tracer of marine-derived resources, given that marine producers are generally enriched in δ^34^S (>15‰), compared to other systems.^38,39^ To further improve resolution of prey origin, we incorporated fatty acid methyl ester (FAME or FA) profiling to apportion aquatic and terrestrial prey sources. Long-chain omega-3 FAs (ω-3), such as eicosapentaenoic acid (EPA, 20:5*n*-3) and docosahexaenoic acid (DHA, 22:6*n*-3), are primarily synthesized by algae and phytoplankton and tend to be concentrated in marine organisms such as cold-water fish, whereas omega-6 FAs (ω-6), such as linoleic acid, are abundant in terrestrial plants, herbivores, and some freshwater fish.^40–43^ Although FA proportions can overlap, relative enrichment patterns (e.g., ω-3/ω-6 ratios) in birds remain strong enough to differentiate dietary pathways because essential FAs cannot be synthesized de novo and must be obtained from the diet.^40–43^ When interpreted alongside other conventional dietary data, stable isotopes and FAs can reduce the risk of misclassification due to overlap and offer a complementary framework for tracing dietary pathways in generalist species, such as bald eagles.^43^ This multivariate approach is important because stable isotope values can vary due to baseline differences among food webs, individuals, habitats, and time periods,^38,44^ limiting their ability to resolve aquatic versus terrestrial origins of contaminants on their own.

Critically, few studies have mechanistically linked individual PFAS and PFAS mixtures to FA profiles. In the Laurentian Great Lakes, elevated ω-3/ω-6 FA ratios in herring gull (*Larus argentatus*) eggs were positively correlated with concentrations of PFOS and other PFAS,^40^ suggesting that increased use of aquatic resources led to high egg PFAS burdens. Analogously, human studies have reported that individuals consuming ω-3 FA-rich diets dominated by marine mammals and seafood tend to have high blood concentrations of long-chain PFCAs, including PFNA and PFUdA.^45,46^ Whether all PFAS are consistently associated with FAs remains uncertain, and comparable assessments in wildlife occupying mixed trophic niches are notably lacking. Bald eagles, with their capacity to integrate contaminants across aquatic and terrestrial food webs, present an ideal model to test whether ω-3 and ω-6 FAs can account for inter and intraregional differences in PFAS exposure.

This study represents the first in-depth assessment of PFAS exposure in bald eagles across multiple regions from the Pacific Northwest and is also one of the first attempts in a wide-ranging predator to apply a multi-tracer approach combining stable isotopes and FAs to contextualize habitat use and PFAS exposure pathways. Our specific objectives were to (1) evaluate spatial patterns in PFAS across a marine–freshwater–terrestrial gradient; (2) examine how PFAS profiles vary with habitat and diet using stable isotope analyses of δ^13^C, δ^15^N, and δ^34^S; and (3) use quantitative FA profiling to compare relative contributions of aquatic vs terrestrial prey across sites. We hypothesized that PFAS levels would vary by habitat and region, reflecting localized point sources in urban areas and long-range transport (LRT) in rural or sparsely populated coastal areas. Given the compound-specific physiochemical properties of PFAS, we further predicted that some PFSAs, notably PFOS, would be linked to more anthropogenic and terrestrial diets, while long-chain PFCAs would be associated with more marine-based diets.

## Materials and Methods

### Sampling Sites and Collections

Sampling locations have been described previously.^43^ Briefly, 89 bald eagle nestlings (*n* = 62 independent nest sites, mean nestling age = 41 d, range = 10–66 d) were sampled from BC, Canada, from 2021 to 2023 (Figure 1). Nest sites were initially located by ground, boat, and aerial surveys and were then prioritized based on the tree structure (i.e., safety), accessibility, land-owner permission, and proximity to urban and industrial pollutant sources. To facilitate spatial comparisons, we divided the study area into eight regions based on established geography, habitat type, and degree of urbanization: Burrard and Howe Sound (*n* = 6), Delta (*n* = 30), Fraser Valley (*n* = 10), North Salish (*n* = 13), South Salish (*n* = 9), Southeast Vancouver Island (*n* = 11), Thompson River (*n* = 6), and the west coast (*n* = 4).^43^ Coastal regions (e.g., Salish Sea, Burrard and Howe Sound, Southeast Vancouver Island) represent predominantly marine-influenced habitats, whereas the Thompson River region reflects more inland and terrestrial environments, and the Fraser River-Delta region represents a semi-urban transition zone. Nest sites located on the west coast of Vancouver Island served as reference sites.

**Figure 1:**
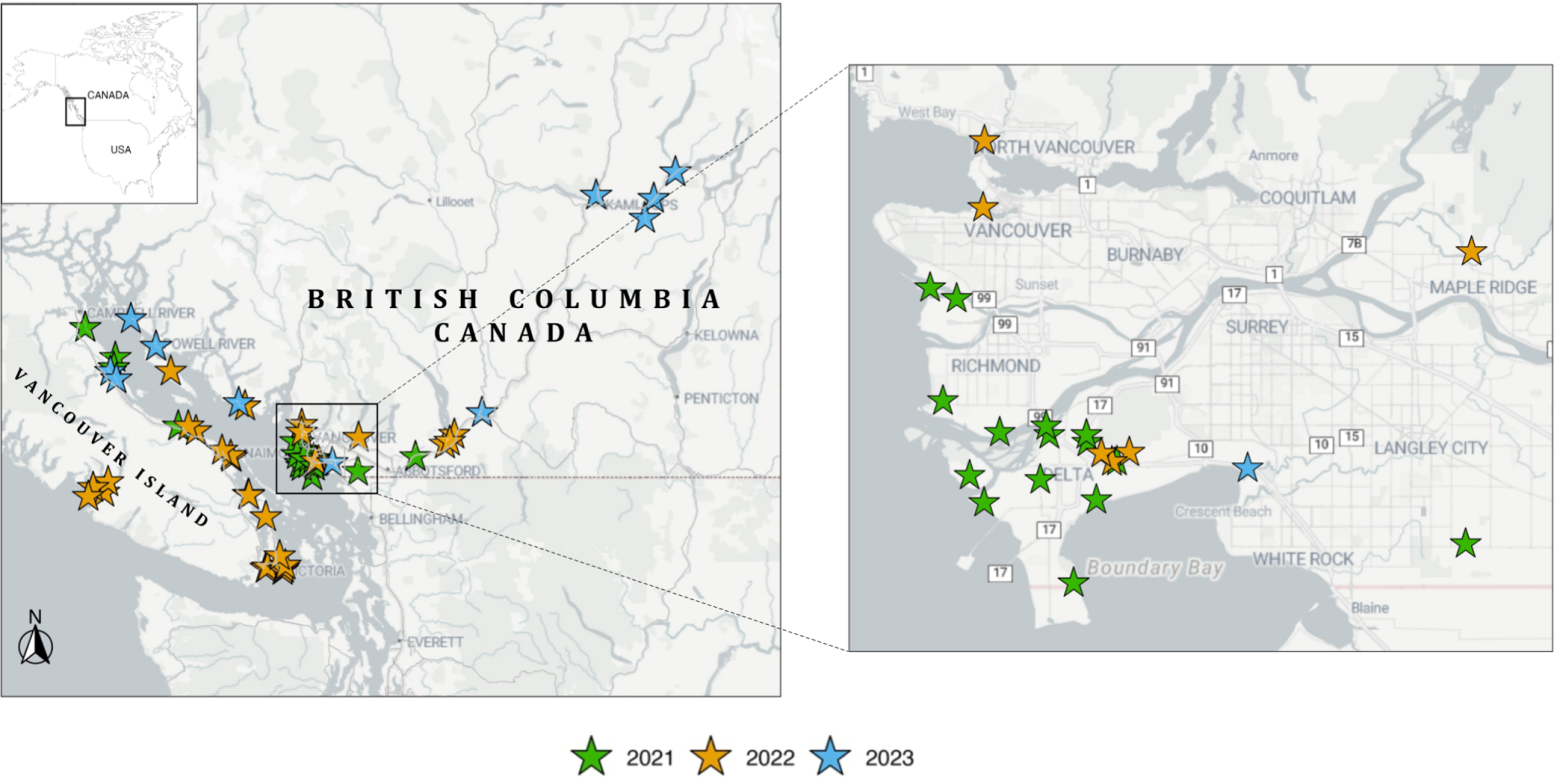
Map of study locations for nestling bald eagle sites (*n* = 89 nestlings; 8 major regions) sampled from the Pacific coast, lower mainland, and interior of British Columbia (BC), Canada, 2021 to 2023. The spatial extent of sampling sites is indicated by the rectangle in the inset (top left corner).

Sampling methods have been described previously.^43^ For each nestling, we used a 21-gauge needle and syringe to obtain up to 24 mL of whole blood from the brachial vein (12 mL per wing). Samples were transferred into ethylenediamine tetraacetic acid (EDTA) vacutainers and kept on ice in the field. By the end of the day, whole blood samples were centrifuged at 3300 rpm for 5 min to separate plasma from cellular fractions. A 500 μL aliquot of plasma from each nestling was transferred into separate polypropylene vials for subsequent analyses. All plasma samples were stored at −20 °C at the National Wildlife Research Centre (NWRC; Ottawa, ON) until processing. Handling, sampling, and banding of bald eagle nestlings were carried out under permits issued by the BC Ministry of Forests, Lands, Natural Resource Operations and Rural Development (SU20-605740, SU22-729698, NA22-704957) and followed guidelines of the Canadian Council on Animal Care (20210118-20JE05).

### Chemical Analyses

Plasma samples were analyzed for a broad standardized suite of 17 PFAS at the NWRC. The full list of Σ_4_PFSAs included PFOS, perfluorohexanesulfonate (PFHxS), perfluorobutanesulfonic acid (PFBS), and perfluorodecanesulfonic acid (PFDS). The full list of Σ_13_ PFCAs analyzed included PFOA, perfluorobutanoic acid (PFBA), perfluoropentanoic acid (PFPeA), perfluorohexanoic acid (PFHxA), perfluoroheptanoic acid (PFHpA), perfluorononanoic acid (PFNA), perfluorodecanoic acid (PFDA), perfluoroundecanoic acid (PFUdA), perfluorododecanoic acid (PFDoA), perfluorotridecanoic acid (PFTriDA), perfluorotetradecanoic acid (PFTeDA), perfluorohexadecanoic acid (PFHxDA), and perfluorooctadecanoic acid (PFODA).

An aliquot of 200 μL of each sample was transferred into a 1.5 mL Eppendorf microcentrifuge tube and spiked with 25 μL of an internal standard solution (ISS; 200 ng/mL in methanol/water, 75:25), prepared as a 10× dilution of the commercial Wellington Laboratories stock solution (MPFAC-MXA; MPFBA, MPFHxA, MPFOA, MPFNA, MPFDA, MPFUdA, MPFDoA, MPFHxS, and MPFOS). Each sample was vortexed and centrifuged at 10,000 rpm for 5 min at room temperature. Approximately 0.5 mL of the supernatant was transferred to a 0.22 μm Nylon centrifuge filter, and each sample was spun for another 5 min at the same speed. The filtrate was then transferred to a 700 μL PFC-free polypropylene plastic autosampler vial and injected into the LC–MS. The 17 PFAS and 9 ISSs were quantified using an Agilent 1260/90 HPLC operating system equipped with a trap column (X-Terra MS C18; 3.5 μm, 2.1 × 100 mm; WATERS 186000404) coupled to a Sciex API 5500 Triple Quadrupole Mass Spectrometer with the TurboSpray ion source in negative polarity using multiple reaction monitoring (MRM) and a column temperature of 50 °C. To ensure optimal separation of individual compounds, an Infinity Lab Poroshell 120 EC-C18; 50 × 3 mm ID, 2.7 μm particle size (Agilent 6999975-302T) was selected for analyses. The sulfonate compounds (PFBS, PFHxS, and PFDS) are reported as the “L” (linear) isomers. PFOS is reported as “Total PFOS” (linear + branched isomers).

### Dietary Tracers

#### Stable Isotope Analyses

SIAs were performed at the Ján Veizer Stable Isotope Laboratory at the University of Ottawa. Samples were freeze-dried and weighed in tin capsules. Blood samples were analyzed for δ^13^C and δ^15^N using a VarioEL Cube Elemental Analyzer (EA: Elementar, Germany) interfaced to a Delta Advantage isotope ratio mass spectrometer (IRMS; Thermo, Germany). The samples were then flash combusted at approximately 1800 °C (Dumas combustion), and the resulting gas products were carried by helium through columns of oxidizing/reducing chemicals optimized for CO_2_ and N_2_. Gas was separated by a “purge and trap” adsorption column and sent to a Conflo IV IRMS (Thermo, Germany) and then to the IRMS. For δ^34^S, blood samples were weighed into tin capsules with tungsten oxide (WO3) and loaded into an Isotope Cube elemental analyzer (EA; Elementar, Germany) in S mode. Released gases were carried by helium through the EA where water was chemically removed, and SO_2_ was separated from CO_2_ and N_2_ by a trap and purge column. SO_2_ gas was then carried into a DeltaPlusXP isotope ratio mass spectrometer (IRMS; Thermo, Germany) via a Conflo IV interface for ^34^S determination. Analytical precision was ±0.3 permil. Details about SIAs and internal standards are provided in Supporting Information.

#### Fatty Acid Analyses

Briefly, 15–20 mg of each plasma sample was weighed, spiked with an IS (5-α-Cholestane), and extracted by sonication. Purified extracts were analyzed for 38 FAs using a capillary gas chromatograph coupled with a flame ionization detector (Agilent Technologies, CA, USA). ∑_5_ Omega-3 FAs comprised a-linolenic acid (ALA), *cis*-11,14,17-eicosatrienoic acid (ETE), *cis*-5,8,11,14,17-eicosapentaenoic acid (EPA), docosapentaenoic acid (DPA), and docosahexaenoic acid (DHA). ∑_7_ Omega-6 FAs comprised linolelaidic acid, linoleic acid, g-linolenic acid (GLA), *cis*-11,14-eicosadienoic acid methyl ester, *cis*-8,11,14-eicosatrienoic acid (DGLA), arachidonic acid (ARA), and *cis*-13,16-docosadienoic acid. Percent lipid determination was achieved gravimetrically. Details about FA analyses are provided in Supporting Information.

### Quality Assurance/Quality Control

QA/QC was assessed using duplicate extractions, solvent blanks (water:methanol 25:75), method blanks, matrix spikes, and second source standards. Three to five solvent blanks were injected at the beginning and end of each set of samples and before and after the calibration standards to monitor cross-contamination. As there is no commercial plasma reference material available, we used 200 μL of distilled water spiked with the IS solution as a method blank, which was extracted with each set of 10 samples. Method accuracy was evaluated by the recoveries of a matrix spike: a 200 μL DI water aliquot was spiked with the IS solution and an SRM solution containing all 17 PFAS and extracted with each set of 10 samples. All samples presented here were corrected for any interference. The recoveries for all PFAS were within the acceptable ranges (80–103%), demonstrating good method accuracy. The relative percent differences (RPDs) between samples extracted and analyzed in duplicate for the results above the method reporting limit (MRL) were all below 20%, demonstrating good method precision. Method detection limits (MDLs) and method reporting limits (MRLs) are listed in Supporting Information Table S1.

### Statistical Analyses

All statistical analyses were performed in R (V4.2) (see Table 2). To reduce sensitivity to compound-specific non-detections and to account for collinearity among individual PFAS, statistical analyses were restricted to PFOS (detected in 100% of nestlings) and on PFAS mixtures (∑_4_ ∑ PFSAs, ∑_13_ PFCAs, ∑_17_ PFAS). Concentrations of PFAS in plasma samples that were not detected or were less than the method detection limit (MDL) were summarized for each region using a Kaplan–Meier (KM) model with the *NADA2* package in R and set to 0 for calculating summary statistics and relative proportions. All PFAS concentrations were expressed in wet weight (ww; ng/mL) and were ln-transformed prior to any statistical analyses.

**Table 1:** Concentrations of Perfluorosulfonic Acid (PFSA) Concentrations (Arithmetic Mean ± Standard Error; ng/mL Wet Weight) in Nestling Bald Eagle Plasma (*n* = 89) Sampled from British Columbia (BC), Canada, 2021 to 2023^b^

sampling region	sample size	lipid (%)	PFOS	PFBS	PFDS	PFHxS	∑_4_ PFSAs^a^
Burrard & Howe	*n* = 6	0.68 ± 0.04 (0.56–0.81)	23.4 ± 9.1 (4.85–53.2)	ND	0.69 ± 0.2 (0.226–1.49)	0.64 ± 0.2 (ND-1.50)	24.7 ± 9.6 (5.12–56.2)
Delta	*n* = 30	0.64 ± 0.04 (0.34–1.2)	40.9 ± 7.3 (2.05–159)	0.70 ± 0.06 (ND-1.56)	0.55 ± 0.1 (ND-4.31)	3.52 ± 1.0 (ND-23.3)	45.6 ± 8.3 (2.95–178)
Fraser Valley	*n* = 10	0.68 ± 0.04 (0.45–0.84)	16.9 ± 3.5 (4.97–41.1)	0.64 ± 0.08 (ND-1.12)	0.30 ± 0.08 (ND-0.820)	1.32 ± 0.7 (0.219–7.30)	18.8 ± 4.2 (5.49–49.2)
North Salish Sea	*n* = 13	0.63 ± 0.02 (0.53–0.75)	11.1 ± 2.7 (2.75–34.3)	0.72 ± 0.1 (ND-1.52)	0.25 ± 0.04 (0.083–0.566)	0.59 ± 0.2 (ND-2.64)	12.3 ± 2.9 (3.42–35.2)
South Salish Sea	*n* = 9	0.81 ± 0.04 (0.61–1.0)	29.7 ± 6.1 (6.96–61.0)	ND	0.70 ± 0.2 (0.099–2.19)	2.06 ± 0.4 (0.565–3.68)	32.5 ± 6.5 (7.90–66.4)
Southeast Van Island	*n* = 11	0.75 ± 0.04 (0.53–1.0)	8.01 ± 0.8 (4.58–12.2)	0.57 ± 0.04 (ND-0.796)	0.25 ± 0.02 (0.173–0.385)	0.33 ± 0.03 (ND-0.50)	8.71 ± 0.9 (4.97–13.6)
Thompson River	*n* = 6	0.70 ± 0.04 (0.59–0.88)	5.75 ± 1.3 (3.04–10.5)	0.74 ± 0.1 (ND-1.20)	0.18 ± 0.04 (0.097–0.370)	0.52 ± 0.1 (ND-1.01)	6.67 ± 1.6 (3.76–11.8)
West Coast	*n* = 4	0.98 ± 0.1 (0.86–1.2)	5.86 ± 1.6 (3.35–10.3)	ND	0.10 ± 0.02 (ND-0.138)	0.46 ± 0.3 (ND-1.20)	6.32 ± 1.9 (3.35–11.6)

a Σ_4_ PFSAs is the sum of PFOS, PFBS, PFDS, and PFHxS.

b Concentrations shown in parentheses are ranges. ‘ND’ = Not detected based on the method detection limit.

**Table 2:** Concentrations of Perfluorocarboxylic Acid (PFCA) Concentrations (Arithmetic Mean ± Standard Error; ng/mL Wet Weight) in Nestling Bald Eagle Plasma (*n* = 89) Sampled from British Columbia (BC), Canada, 2021 to 2023^c^

	sampling region							
analyte	Burrard & Howe (*n* = 6)	Delta (*n* = 30)	Fraser Valley (*n* = 10)	North Salish Sea (*n* = 13)	South Salish Sea (*n* = 9)	Southeast Van Island (*n* = 11)	Thompson River (*n* = 6)	West Coast (*n* = 4)
PFBA	ND	0.144 ± 0.02 (ND-0.468)	0.181 ± 0.06 (ND-0.635)	0.097 ± 0.01 (ND-0.097)	0.118 ± 0.02 (ND-0.165)	ND	ND	ND
PFPeA	ND	ND	ND	0.203 ± 0.02 (ND-0.203)	ND	0.109 ± 0.02 (ND-0.207)	ND	ND
PFHxA	ND	0.287 ± 0.01 (ND-0.287)	0.316 ± 0.03 (ND-0.316)	ND	ND	ND	ND	ND
PFHpA	0.102 ± 0.03 (ND-0.162)	0.121 ± 0.02 (ND-0.392)	0.649 ± 0.5 (ND-5.10)	0.115 ± 0.03 (ND-0.315)	0.175 ± 0.03 (ND-0.262)	0.111 ± 0.02 (ND-0.162)	ND	0.115 ± 0.03 (ND-0.160)
PFOA	0.628 ± 0.2 (ND-0.984)	0.564 ± 0.1 (ND-2.58)	0.941 ± 0.4 (ND-4.32)	0.284 ± 0.04 (ND-0.391)	0.283 ± 0.05 (ND-0.315)	ND	ND	ND
PFNA	1.62 ± 0.30 (0.773–2.49)	2.82 ± 0.28 (0.181–6.02)	2.51 ± 0.71 (0.658–7.37)	2.35 ± 0.33 (0.972–5.05)	1.76 ± 0.26 (0.547–2.97)	2.06 ± 0.19 (1.31–3.17)	1.57 ± 0.4 (0.771–3.35)	2.85 ± 0.89 (1.72–5.48)
PFDA	2.30 ± 0.58 (0.831–4.09)	2.71 ± 0.32 (0.235–8.34)	1.71 ± 0.40 (0.406–3.93)	2.79 ± 0.41 (1.08–5.64)	1.99 ± 0.41 (0.577–4.60)	2.19 ± 0.21 (1.25–3.13)	1.42 ± 0.36 (0.655–3.11)	1.61 ± 0.28 (1.20–2.42)
PFUdA	2.83 ± 0.56 (1.46–5.22)	2.04 ± 0.17 (0.402–4.89)	2.00 ± 0.60 (0.364–6.38)	4.30 ± 0.60 (1.87–10.0)	2.42 ± 0.48 (0.707–4.73)	3.50 ± 0.33 (1.91–5.09)	2.97 ± 0.51 (1.14–4.63)	4.99 ± 1.0 (3.73–8.06)
PFDoA	3.45 ± 1.52 (0.788–8.74)	1.90 ± 0.35 (0.066–7.34)	0.91 ± 0.30 (0.231–3.41)	1.05 ± 0.17 (0.380–2.61)	2.00 ± 0.48 (0.457–5.07)	0.977 ± 0.10 (0.569–1.33)	0.710 ± 0.13 (0.344–1.21)	0.458 ± 0.15 (0.223–0.898)
PFTrDA	2.15 ± 0.66 (0.743–4.73)	0.921 ± 0.12 (0.097–3.36)	0.820 ± 0.29 (0.185–3.29)	1.13 ± 0.13 (0.662–2.25)	1.44 ± 0.27 (0.394–2.78)	1.03 ± 0.10 (0.596–1.55)	1.24 ± 0.34 (0.404–2.72)	1.44 ± 0.31 (1.07–2.37)
PFTeDA	2.14 ± 1.0 (0.413–5.61)	0.788 ± 0.2 (ND-4.94)	0.394 ± 0.1 (0.114–1.52)	0.379 ± 0.08 (0.109–1.06)	1.29 ± 0.3 (0.243–3.08)	0.317 ± 0.05 (ND-0.620)	0.297 ± 0.07 (0.107–0.601)	0.184 ± 0.06 (0.114–0.320)
PFHxDA	0.154 ± 0.05 (ND-0.284)	0.091 ± 0.005 (ND-0.120)	0.114 ± 0.01 (ND-0.114)	ND	0.119 ± 0.02 (ND-0.175)	0.085 ± 0.01 (ND-0.085)	ND	0.085 ± 0.03 (ND-0.100)
PFODA	ND	ND	ND	ND	ND	ND	ND	ND
∑_13_PFCAs^a^	14.92 ± 4.3 (5.78–29.5)	11.66 ± 1.1 (0.981–26.7)	9.74 ± 3.1 (2.33–34.3)	12.14 ± 1.6 (5.19–24.8)	11.31 ± 2.0 (3.14–22.2)	10.14 ± 0.87 (6.07–14.7)	8.21 ± 1.64 (3.42–15.0)	11.67 ± 2.7 (8.25–19.5)
∑_17_PFAS^b^	39.66 ± 14 (12.9–85.7)	57.2 ± 8.7 (3.98–190)	28.5 ± 6.9 (10.1–83.5)	24.5 ± 4.2 (8.61–54.8)	43.8 ± 8.4 (11.0–88.6)	18.9 ± 1.4 (11.0–25.9)	14.9 ± 2.9 (8.30–26.8)	18.0 ± 4.5 (12.0–31.2)

a Σ_13_PFCAs is the sum of all of the PFCAs listed above.

b ∑_17_PFAS is the sum of all of the PFCAs and PFSAs.

c Concentrations shown in parentheses are ranges. ‘ND’ = Not detected based on the method detection limit.

We assessed the normality of residuals by visually inspecting QQ plots and examining residual versus fitted value plots to check for deviations from normality and homoscedasticity. These diagnostics confirmed that the residuals were approximately normally distributed, justifying the use of parametric methods. Dietary tracers, including δ^15^N, δ^13^C, δ^34^S, and FAs (∑ Omega-3 and ∑ Omega-6 FAs) were compared among regions using a Linear Mixed Model (LMM) with a Gaussian distribution and an identity link function (*lme4* package). Each dietary tracer was analyzed separately as the dependent variable with the sampling region as a fixed effect and the nest site as a random effect to account for the non-independence of nestlings sampled from the same nest (i.e., pseudoreplication). To examine relationships between PFAS concentrations and dietary tracers, we first conducted pairwise correlations using Spearman’s rank correlation coefficient, suitable for non-linear and non-parametric data with unequal sample sizes. All *p-*values were computed using the cor.test function, and to control for multiple comparisons and the risk of conducting Type I errors, we applied a false discovery rate (FDR) correction with the Benjamini–Hochberg method. All correlation matrices were visualized using a heatmap.

Relationships between ln-transformed PFAS concentrations and predictor variables were assessed by using LMMs. Fixed effects included nestling age, sex, region, and ∑ Omega3:6 (to represent relative proportions of Omega-3 and Omega-6 FA levels), as well as all three stable isotopes (δ^15^N, δ^13^C, δ^34^S), which capture complementary information on the trophic level, benthic vs pelagic carbon source, and marine vs freshwater/estuarine sulfur source, respectively. Body mass was not included as a variable due to its collinearity with age, while the nest site was included as a random effect. We used the Akaike Information Criterion adjusted for small sample sizes (AIC_C_) with the *MuMIN* package to identify the most parsimonious model. We prioritized models with ΔAIC < 2 and considered model weights, log-likelihood, and conditional *R* ^2^ to evaluate support for each model. When multiple models met these criteria, we used model averaging to assess the relative importance of multiple predictor variables in explaining the variation in the PFAS exposure. Statistical significance was assessed at α = 0.05.

## Results and Discussion

### Dietary Tracers: Stable Isotope Analyses and Fatty Acids

Stable isotopes (δ^13^C, δ^15^N, δ^34^S) and FA profiling were used in a previous assessment of regional dietary patterns in the same nestling bald eagles sampled here.^43^ Generally, coastal and urban bald eagle nestlings from our study exhibited enriched δ^13^C, δ^34^S, and δ^15^N values consistent with mostly marine-derived prey sources, while nestlings from more inland, rural, and landfill sites had depleted stable isotope signatures indicative of freshwater, estuarine, and terrestrial sources^43^ (Figure 2). Based on multivariate and ordination analyses, elevated ∑ Omega-3 FA levels and high ω-3/ω-6 FA ratios in 18% of the studied bald eagle nestlings were consistent with diets dominated by cold-water fish, such as herring, mackerel, and salmon.^29,30,34,43,47^ Conversely, elevated ∑ Omega-6 FA levels and low ω-3/ω-6 FA ratios in 36% of the studied nestlings indicated a greater proportional contribution of none-marine prey items, including terrestrial vertebrates and some freshwater fish.^30,34,43,48^

**Figure 2:**
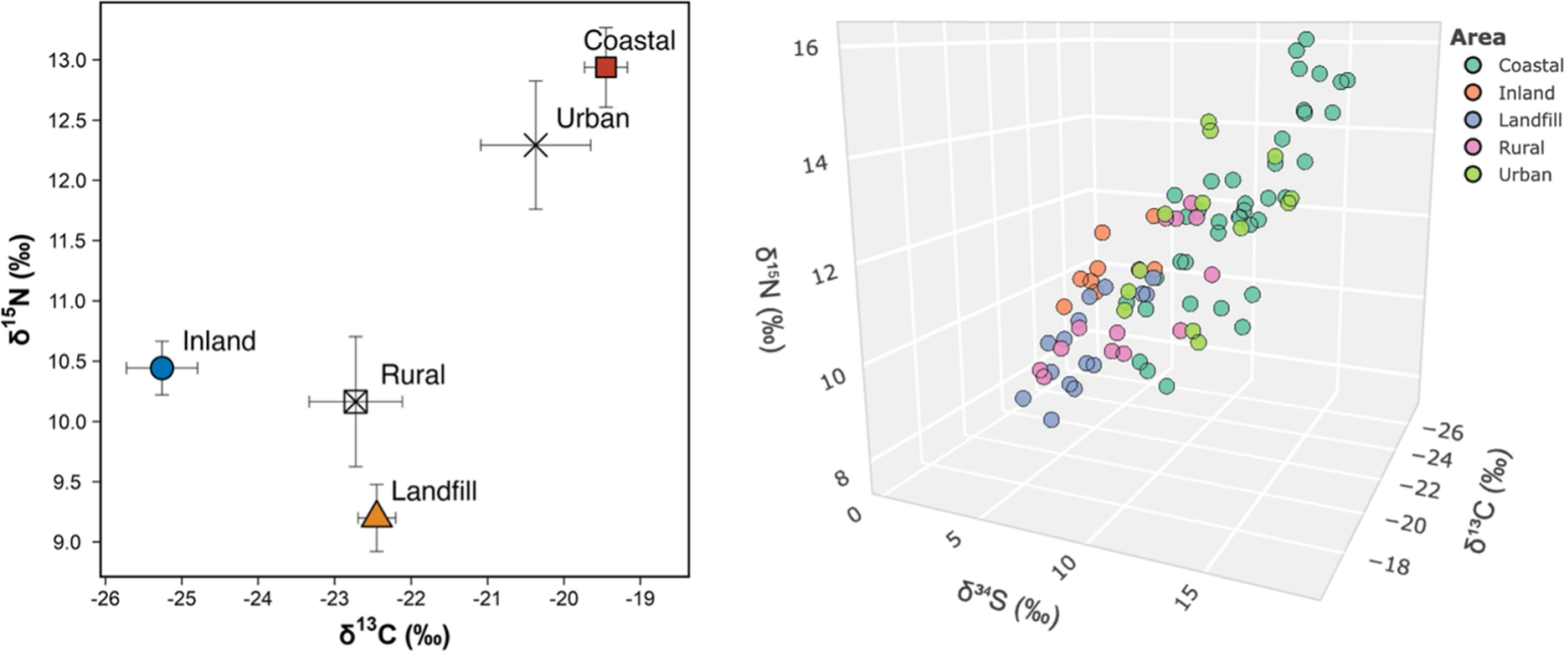
Left plot: Mean δ^15^N (nitrogen) and δ^13^C (carbon) isotope biplot for nestling bald eagle plasma (*n* = 89) sampled from British Columbia (BC), Canada, 2021 to 2023 by habitat type. Error bars represent the standard error. Right plot: Stable isotope 3D plot for δ^15^N, δ^13^C, and δ^34^S (sulfur) values in nestling bald eagle plasma.

Mixed correlations were observed between PFAS and stable isotope values (Figures 3 and 4). PFUdA and PFTrDA were positively correlated with δ^13^C, δ^15^N, and δ^34^S (Figure 3), consistent with exposure through marine-based diets, high-trophic-level foraging, and coastal habitat use. PFOS, PFBS, and PFHxS exhibited negative correlations with δ^13^C, δ^15^N, and δ^34^S (Figure 3). Other PFCAs (e.g., PFNA, PFDA, PFDoA, PFHpA, and PFTeDA) exhibited negligible correlations with stable isotopes (Figure 3), suggesting that stable isotopes do not uniformly predict PFAS exposure in bald eagles and other species.^5,38,40,49^ While some studies have linked trophic position (δ^15^N) and habitat use (δ^13^C, δ^34^S) with PFAS and other organic contaminants in some avian species,^5,10,19,50,51^ our findings suggest this distinction is not consistent across all PFAS, nor is it consistent for the same isotopic tracer, taxa, and geographic region. For instance, a negative correlation between δ^13^C and PFCAs in migratory birds could reflect offshore foraging (depleted δ^13^C signals) and exposure via LRT,^5,50^ while positive correlations between such stable isotopes and PFCAs in nestlings and resident bird species may point to localized sources through enriched δ^13^C prey, such as those observed in the present study.

**Figure 3:**
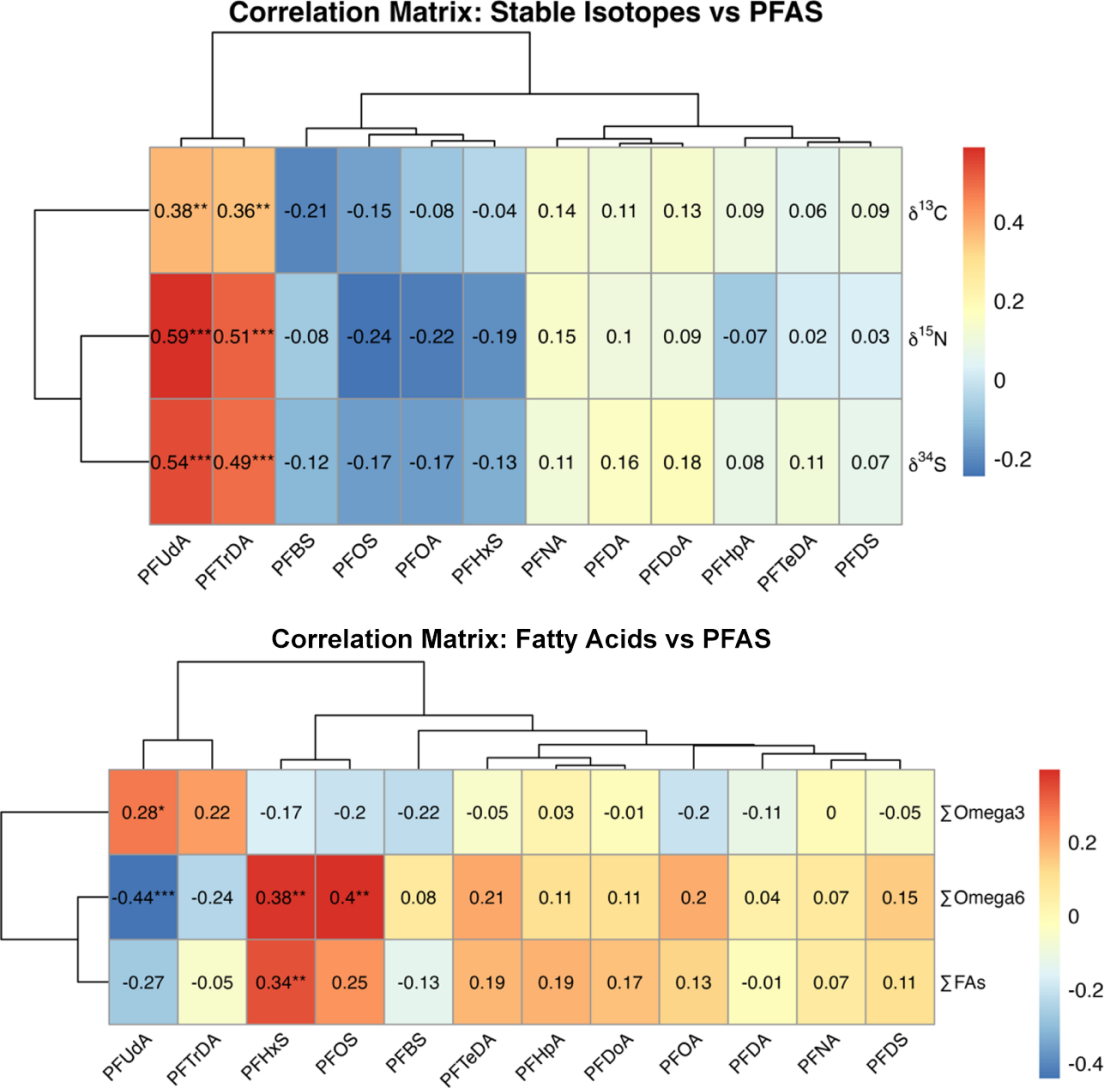
Pairwise correlation matrix illustrating the relationships between stable isotopes of δ^13^C, δ^15^N, and δ^34^S (top) and fatty acids (FAs; bottom) and PFAS concentrations (ng/mL) in nestling bald eagle plasma (*n* = 89) sampled from British Columbia (BC), Canada, 2021 to 2023. Positive (red) and negative (blue) correlations are represented by color intensity and direction. Numbers denote Spearman correlation coefficients. Asterisks denote statistical significance (* = *p* < 0.05, ** = *p* < 0.01, *** = *p* < 0.001).

**Figure 4:**
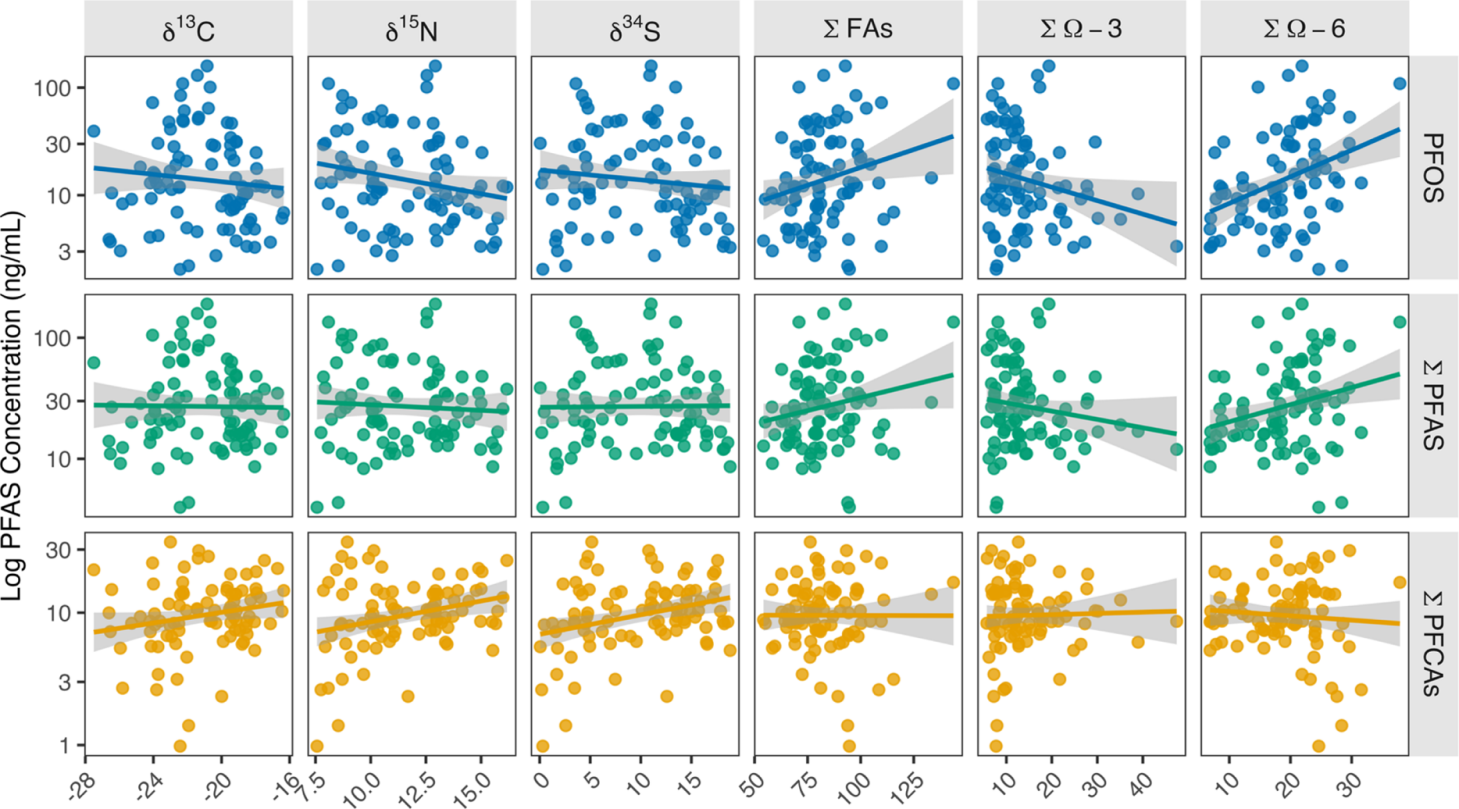
Associations between perfluoroalkyl substance (PFAS) concentrations and dietary tracers, including stable isotopes (first three panels) and fatty acids (last three panels) in nestling bald eagle plasma (*n* = 89) sampled from British Columbia (BC), Canada, 2021 to 2023.

∑ Omega-3 FAs were positively correlated with PFUdA (*r*_s_ = 0.28; *p* < 0.05) and PFTrDA (*r*_s_ = 0.22; Figures 3 and 4), suggesting that these PFCAs were associated with aquatic-based diets, a pattern also observed in herring gull eggs from the Great Lakes^40^ and in northern human communities relying on marine foods.^45,46^ ∑ Omega-6 FAs exhibited significant positive correlations with PFOS (*r*_s_ = 0.40; *p* < 0.01) and PFHxS (*r*_s_ = 0.38; *p* < 0.01; Figure 3), consistent with elevated inputs from terrestrial food sources. Indeed, Kelly et al. (2024) estimated that PFOS biomagnified up to ∼5*×* more in terrestrial food webs compared to aquatic food webs, which the authors attributed to its aqueous solubility, low volatility, protein partitioning coefficients, and rapid elimination in gill-breathing taxa.^1^ Thus, for a given environmental PFOS concentration, birds are expected to attain steady-state body burdens higher than those of fish, even within the same system. That may partly explain why nestlings from the west coast with predominantly fish-based diets had low PFAS levels. However, PFOS exposure in birds is also influenced by the magnitude and proximity of local and non-point sources, which may mask dietary tracer relationships. Therefore, while bald eagles (and their nestlings) feeding on more fish could have had lower PFOS biomagnification than those relying on terrestrial or mammalian prey, greater variability in PFOS/PFAS concentrations is expected among individuals foraging in freshwater and terrestrial environments with more localized contaminant sources.

Although FA profiles can be influenced by abiotic and biotic factors, ω-3 FA availability in Pacific coastal food webs has remained largely stable in recent decades.^41^ As a result, FA profiles in most Pacific birds likely reflect dietary pathways rather than broad environmental shifts and are unlikely to be entirely spurious or artifacts of spatial heterogeneity. In the present study, we detected no significant negative associations between individual FAs and PFAS. Thus, we interpret FAs primarily as dietary tracers, with the observed negative correlations between PFAS and ∑ω-3 FAs and positive associations between PFAS and ∑ω-6 FAs more parsimoniously reflecting dietary sources than contaminant-induced metabolic disruption. Future work could integrate prey sampling along with compound-specific isotopes or fatty acids to better refine the trophic transfer pathways of PFAS in Pacific bald eagles and their prey.

### Perfluoroalkyl Sulfonic Acid Profile

PFOS was detected in 100% of samples and was the most abundantly detected PFSA, constituting 68–100% of ∑_4_ PFSAs and 33–72% of ∑_17_ PFAS (Figure 5) across regions, with PFOS concentrations ranging from 2.1 to 159 ng/mL (median = 12.1; geometric mean = 14.0 ng/mL) (Table 1). Plasma PFOS concentrations varied across regions (F_7,54_ = 4.93; *p* < 0.001), with nestlings from Delta exhibiting up to ∼15*×* higher PFOS concentrations than those from other regions (*p* < 0.001; Table 1). The highest PFOS levels were measured in two female nestlings (from the same nest) in an agricultural area on Westham Island at 159 and 130 ng/mL. The next two highest PFOS levels were 109 ng/mL in a female nestling near a major landfill and 101 ng/mL in a female nestling from a site located less than ∼600–900 m from Vancouver International Airport (YVR). The lowest quantifiable PFOS concentrations (<3 ng/mL) were also measured in nestlings sampled near the Vancouver landfill, suggesting it is not a consistent source of PFOS exposure to bald eagles and other local birds.^52^ Possible reasons for this variability include: (1) intermittent use of landfill-associated food resources by adults during the breeding season;^48^ (2) the consumption of relatively less-contaminated prey from surrounding areas; and (3) landfill management practices, such as reductions in solid waste, improved leachate and gas collection, and the diversion of food waste to a private composting facility.^53^

**Figure 5:**
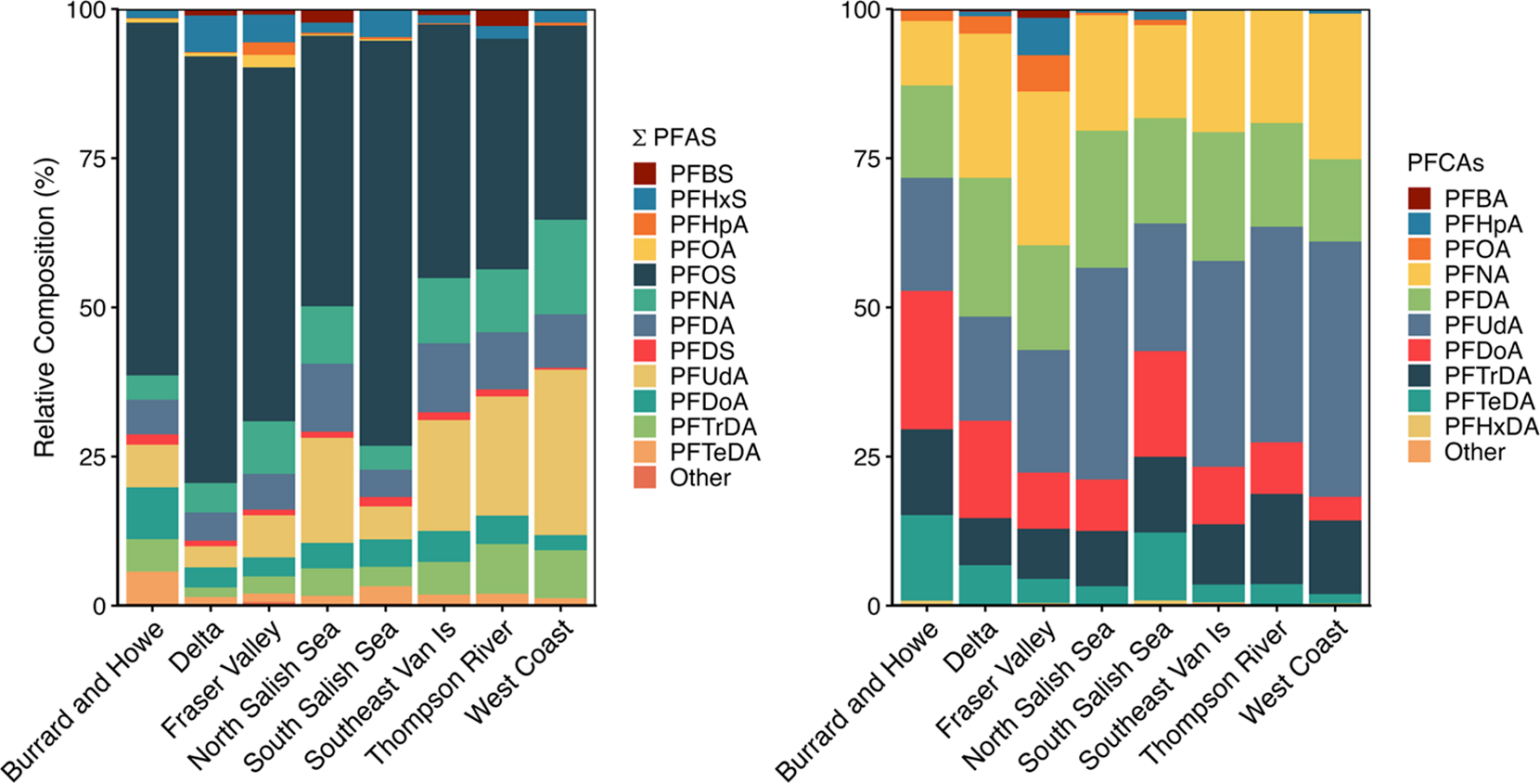
Percent contribution (%) of perfluoroalkyl substances (PFAS) and perfluorocarboxylic acid (PFCA; right) concentrations in nestling bald eagle plasma (*n* = 89) sampled from British Columbia (BC), Canada, 2021 to 2023. With the exception of PFDS and PFDA (both C10) and PFOA and PFOS (both C8), all other PFAS are shown by increasing carbon chain length.

The sources of PFOS and PFHxS in nestlings sampled from Westham Island remain unclear. While Westham Island is dominated by intensive agriculture, assessing the contribution of PFAS-based surfactants from pesticides is challenging due to the proprietary nature of pesticide formulations. As of 2022, eight organofluorine formulants were registered by Canada's Pest Management Regulatory Agency (PMRA), seven of which meet the OECD definition of PFAS.^54^ Whether these inert ingredients degrade into terminal PFSAs and/or PFCAs in soil and biota remain unclear and future studies may be warranted. Another possible pathway of PFAS in this area is the application of WWTP-derived biosolids as fertilizer,^55^ which accounts for ∼43% of biosolid use in Canada.^56^ In fact, a recent study by Gewurtz et al. (2024) revealed that PFOS was widely detected in WWTP-treated biosolids from our study region, with little evidence of temporal decline due to the ongoing use of PFAS in household products and inefficient removal during WWTP processing.^57^ If such biosolids were applied on Westham Island and other nearby agricultural areas, this could represent a more diffuse, albeit persistent, source of PFAS to local biota vial soil uptake and runoff.

The next most abundant PFSAs were PFHxS and PFDS, detected in >80% (0–18% of ∑_4_ PFSAs) and >93% (0–5.3% of ∑_4_ PFSAs) of nestlings, respectively (Figure 5). PFBS was detected in fewer than 45% (0–32% of ∑_4_ PFSAs) of nestlings with similarly low detection frequencies reported elsewhere—less than 25% of bald eagle nestlings sampled across the USA^18,58^ and less than 27% of bald eagle eggs collected from the Great Lakes.^59^ PFHxS occurred at much higher concentrations compared to bald eagle nestlings from the USA.^18,58,60^ In the present study, PFHxS concentrations were up to ∼47 times higher in nestlings sampled near a major wastewater treatment plant (WWTP) and YVR Airport, compared to those from other regions. PFOS and PFHxS were the primary constituents in earlier aqueous film forming foam (AFFF) formulations,^61^ and the elevated levels of these PFSAs in some of those nestlings indicate either historical AFFF usage and/or potentially some ongoing AFFF use in recent years. Similar to our findings, high levels of PFOS (and other PFAS) have been reported in wildlife sampled downstream and/or near major North American airports with known AFFF use.^61,62^

Differences in the occurrence of PFDS, PFBS, and PFHxS may also be linked to regulatory actions and physiochemical properties. For instance, although all three PFSAs were phased out by 3 M in 2002, PFHxS and its salts are currently not regulated in Canada under the Prohibition of Certain Toxicity Substances Regulations (PCTSR). In addition, the low detection frequencies for short-chain PFSAs may stem from their low log *K*_OW_ values and high solubilities, consequently limiting their bioavailability in higher trophic level consumers.^1^ Accordingly, PFBS was unquantifiable in various seabird eggs sampled along the Pacific coast between 1973 and 2019 ^5,25,26^ and also showed evidence of trophic dilution in the food web of an urban population of a terrestrial raptor, the Cooper’s Hawk (*Astur cooperii*), from our region.^13^

PFOS concentrations in Pacific bald eagles have not been previously reported, preventing direct comparisons. Nonetheless, the PFOS plasma concentrations here were substantially lower than the mean PFOS plasma levels previously reported in bald eagle nestlings across midwestern USA, including the Mississippi and St. Croix Rivers between 2006 and 2011 (78–800 ng/mL),^18^ several Wisconsin watersheds between 2011 and 2017 (24–564 ng/mL),^58^ and various inland and nearshore sites spanning multiple U.S states between 1990 and 1993 (330 ng/mL)^63^ and across the Great Lakes between 2006 and 2015 (20–571 ng/mL).^60^ Elevated PFOS concentrations in bald eagle nestlings from midwestern states have been widely attributed to historical PFOS releases from 3M’s primary manufacturing site in Cottage Grove, Minnesota, along with other regional sources such as AFFF use.^18,61^ The PFOS concentrations in midwestern bald eagle nestlings are also 1–2 orders of magnitude greater than those observed in our Pacific coast nestlings of comparable age, a difference unlikely to be explained by diet or physiology alone and more consistent with historical inputs. Moreover, PFOS, its salts, and precursors were never manufactured in Canada and the comparatively lower degree of urbanization and population density in BC (relative to most US states) likely further contributed to the lower PFOS emission history and exposure profile observed in Pacific bald eagle populations.

In further comparisons, PFOS plasma concentrations measured in this study were higher than those reported in the nestlings of other species. PFOS plasma concentrations here were up to ∼6.5*×* higher than those observed in some white-tailed eagle (*Haliaeetus albicilla*) nestlings and northern goshawk (*Accipiter gentilis*) nestlings across Europe between 2010 and 2016 ^49,64^ and up to ∼3.5*×* higher than those in various gull chicks from France between 2016 and 2019.^20^ PFOS concentrations here were also comparable to, and in some cases, up to ∼1.3*×* higher than those measured in peregrine falcon (*Falco peregrinus*) nestlings sampled across urban and rural regions of eastern Canada in 2016 and 2018.^19^ These interspecies comparisons are particularly noteworthy, as PFAS restrictions implemented in the early 2000s should have resulted in reduced exposure across most wildlife populations. Accordingly, inter and intraspecific variation in PFAS exposure among nestlings likely reflects a complex interplay of factors, including proximity to local sources, transport pathways (i.e., slow ocean transport vs rapid atmospheric transport), maternal transfer, diet, in vivo biotransformation, and tissue-specific protein binding affinities that mediate PFAS sorption and retention.^1,12,14,16^

### Perfluoroalkyl Carboxylic Acid Profile

PFNA, PFDA, PFUdA, PFDoA, and PFTrDA were detected in 100% of samples (Supporting Information Table S2). The dominant PFCAs detected were PFUdA > PFNA > PFDA, comprising 32–84% of ∑_13_ PFCAs, although proportions varied by region (Figure 5). PFUdA, PFNA, and PFDA were the most frequently detected PFCAs in most nestlings, with concentrations of these three PFCAs ranging from 0.36 to 10, from 0.18 to 7.4, and from 0.24 to 8.3 ng/mL, respectively, comprising 32–84% of ∑_13_ PFCAs (Figure 5). Nestlings from Burrard Inlet & Howe Sound and the South Salish Sea had PFCAs that were dominated by PFDoA and PFUdA, which accounted for 72% and 39% of ∑_13_ PFCAs, respectively, with concentrations ranging from 0.46 to 8.7 ng/mL. Similarly, double-crested cormorant eggs collected from the South Salish Sea had higher levels of PFDoA and PFUdA compared to offshore seabird species, with marginal increases from 1985 to 2019 attributed to urban/industrial influences from the nearby Victoria and Vancouver harbors.^5^ The heavy industrialization in those areas may explain why nestlings from the Salish Sea had more quantifiable PFCA analytes (>10) compared to conspecifics in less urbanized areas. PFOA levels were generally below detection limits and occurred in only 24% of samples. Short-chain PFCAs, such as PFBA, PFHxA, PFODA, and PFPeA, were also unquantifiable or detected at low concentrations (<0.6 ng/mL), possibly reflecting their low protein partitioning capacities and rapid elimination kinetics in birds.^1,16^

The PFCA patterns observed in the studied bald eagle nestlings contrast with those reported in some species, reflecting differences in manufacturing and transport processes. For example, PFUdA, PFNA, and PFDA were the primary PFCAs detected in bald eagle nestlings in the USA,^18,58,60^ peregrine falcon nestlings in eastern Canada,^19^ and white-tailed eagle nestlings in northern Europe.^65^ By contrast, great tit (*Parus major*), blue tit (*Cyanistes caeruleus*), and tree swallow (*Tachycineta bicolor*) nestlings sampled near various 3 M PFAS facilities were dominated by PFOS and several even-chain PFCAs, including PFOA, PFDoDA, and PFTeDA,^61,66^ possibly reflecting emissions from telomerization-based production, whereas odd and mixed-chain PFCA profiles are consistent with legacy electrochemical fluorination sources.^3^ Elevated odd/even PFCA ratios and the dominance of long-chain PFCAs in bald eagles and other Pacific wildlife may also point to precursor degradation (e.g., fluorotelomer alcohols) as a key pathway for PFCA exposure.^4,67–69^

Following the voluntary phase-out of PFCA production and emissions between 2010 and 2015,^70^ several seabird species from the northeastern Pacific, including Leach’s storm-petrels (*Hydrobates leucorhous*) and rhinoceros auklets (*Cerorhinca monocerata*), exhibited rapid declines in egg ∑ PFCA concentrations, with annual decreases of ∼5–12% observed over the past few decades.^5,17,25^ The rapid response of PFCAs in those species is suggestive of atmospheric transport since PFCA emissions from direct sources can take several years to reach the Pacific Ocean before such a response to emissions occurs.^22,23^ By contrast, glaucous-winged gulls breeding on Mandarte, Mitlenatch, and Cleland Islands (all located in close proximity to some of our eagle nest sites) showed linear egg ∑ PFCA increases (35–44% annually) between 2008 and 2012, followed by no significant trends between 2018 and 2022.^26^ Those PFCA trends were offset by compound-specific increases (e.g., PFNA) and were linked to dietary shifts to more marine-based prey, such as fish, based on egg stable isotope signatures.^26^ Therefore, while regulatory efforts have resulted in measurable PFAS declines in some Pacific wildlife, continued emissions, LRT, and dietary exposure remain challenges for contaminant recovery at the ecosystem level, reinforcing the importance of targeted PFAS surveillance monitoring.

### Effect of Nestling Age on PFSAs and PFCAs

Model averaging identified the sampling region and nestling age as the most consistent predictors of plasma PFAS concentrations (Supporting Information Table S3). Across the region, log-PFOS concentrations increased with nestling age by 2.3%/*d* (*t*_86_ = 2.56; *R* ^2^ = 0.91; *p* < 0.05) (Supporting Information Figure S1 and Supporting Information Table S4). PFDS followed a similar pattern, increasing by 2%/*d* (*t*_80_ = 2.75; *R* ^2^ = 0.92; *p* < 0.01), whereas PFHxS and PFBS exhibited weaker relationships with age, increasing by 1.2%/*d* (*t*_59_ = 1.15; *R* ^2^ = 0.97; *p* = 0.26) and 0.13%/*d* (*t*_27_ = 0.21; *R* ^2^ = 0.34; *p* = 0.84), respectively. Most of the PFSA age increases were driven by nestlings from Delta, where log-PFOS and log-∑_4_ PFSA concentrations increased by 4.3–4.5%/*d* (*t*_30_ = 2.86; *R* ^2^ = 0.94; *p* < 0.01), exceeding the rates at all other locations.

Similarly, model-averaged results from a suite of candidate LMMs identified nestling age as a significant predictor for ∑_13_ PFCA plasma concentrations (Supporting Information Tables S3 and S5), increasing by 1.61%/*d* across the region (*t*_86_ = 2.87; *R* ^2^ = 0.90; *p* < 0.01). The PFCA increases were largely driven by C_9_–C_14_ PFCAs, particularly PFUdA, PFDoA, and PFTrDA, each increasing by 1.45–2.20%/*d* across the region (Supporting Information Figure S2). PFHpA slightly decreased with age (*t*_43_ = −1.61; *p* = 0.12), while PFOA remained relatively constant (*t*_15_ = 0.14; *p* = 0.89) with age (Supporting Information Figure S2).

The age-related accumulation rates for most PFSAs and PFCAs observed here were generally within the range of those reported in other bald eagle nestlings^58,60^ and white-tailed eagle nestlings.^49^ Differences in age-related PFAS accumulation rates are presumably a function of exposure duration, the developmental age span studied, and bioaccumulation potential, as shorter-chain PFAS like PFBS are more rapidly excreted in birds, compared to their longer-chain counterparts.^1^ The increase in PFAS concentrations with age also suggests that some bald eagle nestlings could be fed with relatively more PFAS-contaminated prey items as they grow, either due to dietary shifts, increased feeding frequency (i.e., volume of food), and/or higher energy needs,^34^ since we would have expected some growth dilution to occur. However, the present data set cannot distinguish among these mechanisms, and these explanations should be interpreted as plausible contributors rather than definitive drivers. Notably, previous work on polybrominated diphenyl ethers (PBDEs) and alternative halogenated flame retardants (AHFRs) in the same nestlings did not report analogous age-dependent accumulation patterns,^43^ suggesting that PFAS toxicokinetics in bald eagles (and their nestlings) differs from those of some legacy organic contaminants.

## Toxicological Implications

Recent studies on raptor nestlings and other species from our study region suggest that early biological effects may occur at low PFAS concentrations. For instance, Hansen et al. (2020) reported that plasma concentrations of several long-chain PFCAs (PFUdA, PFDoDA, PFTrDA; 1.2–13.4 ng/g ww) and PFHxS (0.1–3.5 ng/g ww) were negatively associated with leukocyte coping capacity (LCC) in white-tailed eagle nestlings, indicating potential immunomodulation.^74^ Similarly, King et al. (2023) observed significant positive correlations between hepatic PFHpA concentrations (range: < MDL to 0.64 ng/g ww) and cytochrome P450 gene expression in wild-collected rhinoceros auklet embryos from the northeastern Pacific,^75^ with PFCA concentrations comparable to those measured in our study. Collectively, these findings support the relevance of sublethal end points and suggest that even low-level PFAS exposure may elicit early physiological responses in some species from our study region, including bald eagles.

The ecotoxicological effects of PFAS in raptors, particularly in bald eagle nestlings, nonetheless remain poorly characterized. Current regulatory thresholds, such as the Canadian Federal Environmental Quality Guideline (FEQG) and the toxicity reference value (TRV) for PFOS in bird eggs and plasma (1900 ng/mL and 1700 ng/mL, respectively),^71,72^ are several orders of magnitude higher than the concentration detected in our nestlings. Elliott et al. (2019) applied ToxCast high-throughput screening data and reported exposure–activity ratios (EARs) exceeding 1 for PFOS and other PFAS in the majority of bald eagle nestlings sampled in the Midwestern U.S., suggesting potential impacts on metabolic, behavioral, developmental, and cardiac pathways.^73^ However, PFOS/PFAS concentrations in that study (7.5–4200 ng/mL) were substantially higher than those observed here and therefore may not be ecologically relevant. Future work could build on this framework by combining field and lab exposure PFAS assessments with molecular and biomarker assays to identify sublethal effects.

## Supplementary Material



## Notes

Additional details for stable isotope and fatty acid (FA) analyses, method detection limits (MDLs), method reporting limits (MRLs), frequency of detections, parameter estimates for age-dependent PFAS bioaccumulation, and a geospatial analysis of PFAS concentrations (PDF)
