# Supplementary material for: Using a Multi-Tracer Approach to Examine Perfluoroalkyl Substance Sources and Dietary Exposure Pathways in Pacific Bald Eagles
Source: Environ Sci Technol. 2026 Mar 17;60(12):9530–42. doi: 10.1021/acs.est.5c15624 (PMC13045011; doi:10.1021/acs.est.5c15624)
Supplement: Supplementary file 1 [file es5c15624_si_001.pdf]

Using a multi-tracer approach to examine perfluoroalkyl substance (PFAS) sources and dietary exposure pathways in Pacific Bald Eagles

Robert Kesic,[a] John E. Elliott,[a] Myles Lamont,[b] Sandi L. Lee,[a] Kimberly M. Cheng,[c] France Maisonneuve[d]

[a]*Environment and Climate Change Canada, Wildlife Research Division, Delta, British Columbia, Canada, V4K 3N2: rkesicc@gmail.com; john.elliott@ec.gc.ca; sandi.lee@ec.gc.ca*

[b]*TerraFauna Wildlife Consulting, Surrey, British Columbia, Canada, V3Z 9R9: myles@terrafauna.ca*

[c]*University of British Columbia, Applied Animal Biology, Vancouver, British Columbia, Canada, V6T 1Z4: kimberly.cheng@ubc.ca*

[d]*Environment and Climate Change Canada, National Wildlife Research Centre (NWRC), Ottawa, Ontario, K1S 5R2: france.maisonneuve@ec.gc.ca*

Supporting Information

Pages: 10

This Supporting Information (SI) is 10 pages and includes method details for stable isotope and fatty acid (FA) analyses, method detection limits (MDLs), method reporting limits (MRLs), frequency of detections, parameter estimates for age-dependent PFAS bioaccumulation, and a geospatial analysis of PFAS concentrations in plasma samples from 89 bald eagle (*Haliaeetus leucocephalus*) nestlings sampled between 2021 and 2023 across British Columbia, Canada.

Methods

Stable isotope analyses (SIAs)

The internal standards used for $\delta^{13}C$ (in ‰) included: C-51 Nicotiamide (0.07, -22.95), C-52 mix of ammonium sulphate + sucrose (16.58, -11.94), C-54 caffeine (-16.61, -34.46), and a blind standard C-55: L-glutamic acid (-3.98, -28.53). These standards cover the natural range, and the data is reported in Delta notation δ, with units in per mil (‰) and are expressed as:

$$\delta X = \frac{(R_{sample} - R_{standard})}{R_{standard} \times 1000}$$

where δX is ^{15}N, ^{13}C, or ^{34}S, and R is the ratio of the abundance of the heavy to the light isotope in the plasma sample. All $\delta^{15}N$ is reported as ‰ vs. AIR and normalized to internal standards calibrated to international standards IAEA-N1(+0.4‰), IAEA-N2(+20.3‰), USGS-40(-4.52‰) and USGS-41(47.57‰). All $\delta^{13}C$ data is reported as ‰ vs. V-PDB and normalized to internal standards calibrated to international standards IAEA-CH-6(-10.4‰), NBS-22(-29.91‰), USGS-40(-26.24‰) and USGS-41(37.76‰). All $\delta^{34}S$ data is reported as ‰ vs. V-CDT and normalized to an internal standard calibrated to international sulphide standards: IAEA-S1 (-0.3‰), IAEA-S2 (22.7‰), and IAEA-S3 (-32.6‰). The results of the blind STD C-55, L-glutamic acid and the in-house reference material (SRM W030460-02: 2003 DCCO ERM Freeze-dried), were within the acceptable limits. The results of the blind STD S-6 and the in-house reference material (SRM W103300-01 1989 Undiluted HERG ERM Freeze-dried) were also within the acceptable limits.

Fatty acid (FA) analyses

Approximately 15-20 mg of freeze-dried plasma was accurately weighed and spiked with an internal standard (5-α-Cholestane). Lipids were extracted using ultrasonication in 50:50 chloroform:methanol with 1% butylated hydroxytoluene. Lipid extracts were dissolved in toluene and 1% sulfuric acid in methanol and incubated overnight in a heated (50°C) water batch shaker. Following neutralization using 1% potassium carbonate, FAME derivatives were solvent exchanged to hexane for instrumental analysis. Purified sample extracts were analyzed for 38 FAMEs using a capillary GC (Agilent 6890N, Agilent Technologies, CA, USA) coupled with a flame ionization detector. Instrument configuration was: split injection (100:1); SP-2560 Supelco column 100m x 0.25mm internal diameter x 0.20mm; oven program 140 °C (hold 5 min) to 240 °C at 4 °C/min, hold for 12 min; helium carrier gas, 1.2mL/min; flame ionization detector at 260 °C; injector at 260 °C; total run time = 42 min/sample. Results were recovery corrected and corrected for background contamination by subtracting the concentration values of the associated method blank. One herring gull egg in-house reference material diluted and freeze-dried was worked up simultaneously with each extraction batch. Where possible, a duplicate extraction and injection was selected in order to monitor extraction and instrumental precision.

The full list of FAs analyzed included: Butyric Acid, Caproic Acid, Caprylic Acid, Capric Acid, Undecanoic Acid, Lauric Acid, Tridecanoic Acid, Myristic Acid, Myristoleic Acid, Pentadecanoic Acid, cis-10-Pentadecanoic Acid, Palmitic Acid, Palmitoleic Acid, Heptadecanoic Acid, cis-10-Heptadecanoic Acid, Stearic Acid, Elaidic Acid, Oleic Acid, Linolelaidic Acid, Linoleic Acid, Arachidic Acid, g-Linolenic Acid (GLA), cis-11-Eicosenoic acid, a-Linolenic Acid (ALA), Heneicosanoic Acid, cis-11,14-Eicosadienoic Acid Methyl Ester, Behenic Acid, cis-8,11,14-Eicosatrienoic Acid (DGLA), Erucic Acid, cis-11,14,17-Eicosatrienoic Acid (ETE), Arachidonic Acid (ARA), Tricosanoic Acid, cis-13,16-Docosadienoic Acid, Lignoceric Acid,

cis-5,8,11,14,17-Eicosapentaenoic Acid (EPA), Nervonic Acid, Docosapentaenoic Acid (DPA), and Docosahexaenoic Acid (DHA).

Final FA results were recovery corrected and also corrected for background contamination by subtracting the concentration values of the associated method blank. One herring gull egg in-house reference material (HERG-QC-1989), diluted and freeze-dried (K09-21769-00-04), was worked up simultaneously with each extraction batch of samples. Where possible, a duplicate extraction (DUP1) and injection (DUP2) was selected in order to monitor extraction and instrumental precision for all FA analyses.

SI Table 1: Method detection limits (MDL) and method reporting limits (MRL) for PFAS in bald eagle (*Haliaeetus leucocephalus*) nestling plasma (n = 89) from 2021-2023 across British Columbia (BC), Canada. MDLs and MLOQs are expressed in ng/mL. 'n.d' = not detected.

	**Abbreviation**	**Compound**	MDL	MRL
**Sulfonic acids**	PFOS	Perfluorooctane sulfonate	0.17	0.51
	PFBS	Perfluorobutane sulfonate	0.36	1.08
	PFDS	Perfluorodecane sulfonate	0.07	0.20
	PFHxS	Perfluorohexane sulfonate	0.19	0.57
**Carboxylic acids**	PFBA	Perfluorobutanoic acid	0.09	0.28
	PFPeA	Perfluoropentanoic acid	0.08	0.24
	PFHxA	Perfluorohexanoic acid	0.07	0.20
	PFHpA	Perfluoroheptanoic acid	0.08	0.23
	PFOA	Perfluorooctanoic acid	0.27	0.80
	PFNA	Perfluorononanoic acid	0.09	0.28
	PFDA	Perfluorodecanoic acid	0.07	0.22
	PFUdA	Perfluoroundecanoic acid	0.07	0.21
	PFDoA	Perfluorododecanoic acid	0.06	0.17
	PFTrDA	Perfluorotridecanoic acid	0.06	0.17
	PFTeDA	Perfluorotetradecanoic acid	0.10	0.29
	PFHxDA	Perfluorohexadecanoic acid	0.08	0.23
	PFODA	Perfluorooctadecanoic acid	0.12	0.36

The MDLs were determined by calculating the standard deviation (SD) between 8 replicates of a low spike matrix sample that went through the whole procedure. Each MDL corresponds to 3xSD. We established the MRLs as 5x the MDLs. Calibration accuracy was evaluated by using a second source standard solution (solution from NCP-AMP14 PT that contained 10 of the 17 PFAS); aliquots were analyzed with each set of samples to monitor day to day variation.

SI Table 2. Frequency of detection for PFAS in bald eagle (*Haliaeetus leucocephalus*) nestling plasma sampled from British Columbia (BC), Canada, 2021 to 2023.

	PFAS	Compound	*n* Censored	*n* Detected	Detection Frequency (%)
**Sulfonic acids**	PFOS	Perfluorooctane sulfonate	0	89	100
	PFBS	Perfluorobutane sulfonate	49	40	45
	PFDS	Perfluorodecane sulfonate	6	83	93
	PFHxS	Perfluorohexane sulfonate	18	71	80
**Carboxylic acids**	PFBA	Perfluorobutanoic acid	72	17	19
	PFPeA	Perfluoropentanoic acid	84	5	5.6
	PFHxA	Perfluorohexanoic acid	87	2	2.2
	PFHpA	Perfluoroheptanoic acid	43	46	52
	PFOA	Perfluorooctanoic acid	67	22	25
	PFNA	Perfluorononanoic acid	0	89	100
	PFDA	Perfluorodecanoic acid	0	89	100
	PFUdA	Perfluoroundecanoic acid	0	89	100
	PFDoA	Perfluorododecanoic acid	0	89	100
	PFTrDA	Perfluorotridecanoic acid	0	89	100
	PFTeDA	Perfluorotetradecanoic acid	3	86	97
	PFHxDA	Perfluorohexadecanoic acid	72	17	19
	PFODA	Perfluorooctadecanoic acid	89	0	0

SI Table 3. Akaike Information Criterion (AIC) model selection for PFAS concentrations in nestling bald eagle (*Haliaeetus leucocephalus*) plasma (n = 89) sampled from British Columbia (BC), Canada, 2021 to 2023. Models shown below are $\Delta AIC_C < 2$ and are ranked by increasing AIC_C. Predictor variables in bold are statistically significant ($p < 0.05$). Nest site was included as a random effect to avoid pseudo-replication. 'Df' = Degrees of freedom.

**Model**	**Df**	**Log Likelihood**	**AIC_C**	**ΔAIC_C**	**Weight**
PFOS					
PFOS ~ **age + region**	11	-80.97	187.37	0.00	0.30
PFOS ~ **age** + δ^{13}C + **region**	12	-79.73	187.56	0.19	0.27
PFOS ~ **age +** **δ^{13}C** + δ^{15}N + **region**	13	-78.90	188.66	1.29	0.16
PFOS ~ **age + region** + sex	12	-80.29	188.68	1.31	0.16
PFOS ~ **age** + δ^{13}C + **region** + sex	13	-79.19	189.24	1.87	0.12
$\sum_4$PFSAs					
$\sum_4$PFSAs ~ **age + region**	11	-78.27	181.97	0.00	0.38
$\sum_4$PFSAs ~ **age** + δ^{13}C + **region**	12	-77.38	182.87	0.91	0.24
$\sum_4$PFSAs ~ **age + region** + sex	12	-77.44	182.99	1.03	0.23
$\sum_4$PFSAs ~ **age** + Omega3:6 + **region**	12	-77.86	183.83	1.87	0.15
$\sum_{13}$PFCAs					
$\sum_{13}$PFCAs ~ **age** + **δ^{15}N** + Omega3:6 + sex	7	-53.82	123.02	0.00	0.17
$\sum_{13}$PFCAs ~ **age** + δ^{15}N + sex	6	-55.22	123.47	0.45	0.14
$\sum_{13}$PFCAs ~ **age** + **δ^{15}N** + Omega3:6	6	-55.25	123.53	0.51	0.13
$\sum_{13}$PFCAs ~ **age** + δ^{13}C + **δ^{15}N** + Omega3:6	7	-54.24	123.86	0.84	0.11
$\sum_{13}$PFCAs ~ **age** + sex	5	-56.85	124.42	1.41	0.08
$\sum_{13}$PFCAs ~ **age** + δ^{13}C + **δ^{15}N** + Omega3:6 + sex	8	-53.31	124.43	1.41	0.08
$\sum_{13}$PFCAs ~ **age** + δ^{15}N	5	-56.96	124.65	1.63	0.08
$\sum_{13}$PFCAs ~ **age** + δ^{34}S + sex	6	-55.87	124.76	1.75	0.07
$\sum_{13}$PFCAs ~ **age** + δ^{13}C + **δ^{15}N**	6	-55.91	124.84	1.82	0.07
$\sum_{13}$PFCAs ~ **age** + δ^{13}C + δ^{15}N + sex	7	-54.73	124.84	1.83	0.07

SI Table 4. Parameter estimates for linear mixed effects models evaluating age-dependent PFOS bioaccumulation in nestling bald eagle (*Haliaeetus leucocephalus*) plasma (n = 89) sampled from British Columbia (BC), Canada, 2021 to 2023. Nest site was included as a random effect to avoid pseudo-replication. Stars indicate significance: * $p < 0.05$, ** $p < 0.01$, *** $p < 0.001$.

**Predictor**	**Estimate**	**Std. Error**	**df**	**t value**	**p value**
Intercept	1.19	0.545	67.5	2.18	0.033 *
Age	0.028	0.0078	77.2	3.62	0.00052 ***
Delta	0.916	0.423	54.6	2.16	0.035 *
Fraser Valley	0.429	0.477	55.7	0.90	0.373
North Salish Sea	-0.033	0.475	54.9	-0.07	0.944
South Salish Sea	1.06	0.493	56.2	2.16	0.035 *
Southeast Vancouver Island	-0.406	0.470	54.7	-0.87	0.391
Thompson River	-0.793	0.540	54.3	-1.47	0.148
West Coast	-0.816	0.545	56.3	-1.50	0.140

SI Table 5. Parameter estimates for linear mixed effects models evaluating age-dependent $\sum_{13}$PFCA bioaccumulation in nestling bald eagle (*Haliaeetus leucocephalus*) plasma (n = 89) sampled from British Columbia (BC), Canada, 2021 to 2023. Nest site was included as a random effect to avoid pseudo-replication. Stars indicate significance: * $p < 0.05$, ** $p < 0.01$, *** $p < 0.001$.

**Predictor**	**Estimate**	**Std. Error**	**df**	**t value**	**p value**
Intercept	0.823	0.398	83.9	2.07	0.042 *
Age	0.015	0.0054	83.5	2.83	0.0058 **
$\delta^{15}N$	0.076	0.0318	82.2	2.40	0.0185 *
Omega3:6	-0.104	0.0631	84.0	-1.64	0.104
Sex	0.117	0.0694	36.5	1.68	0.101

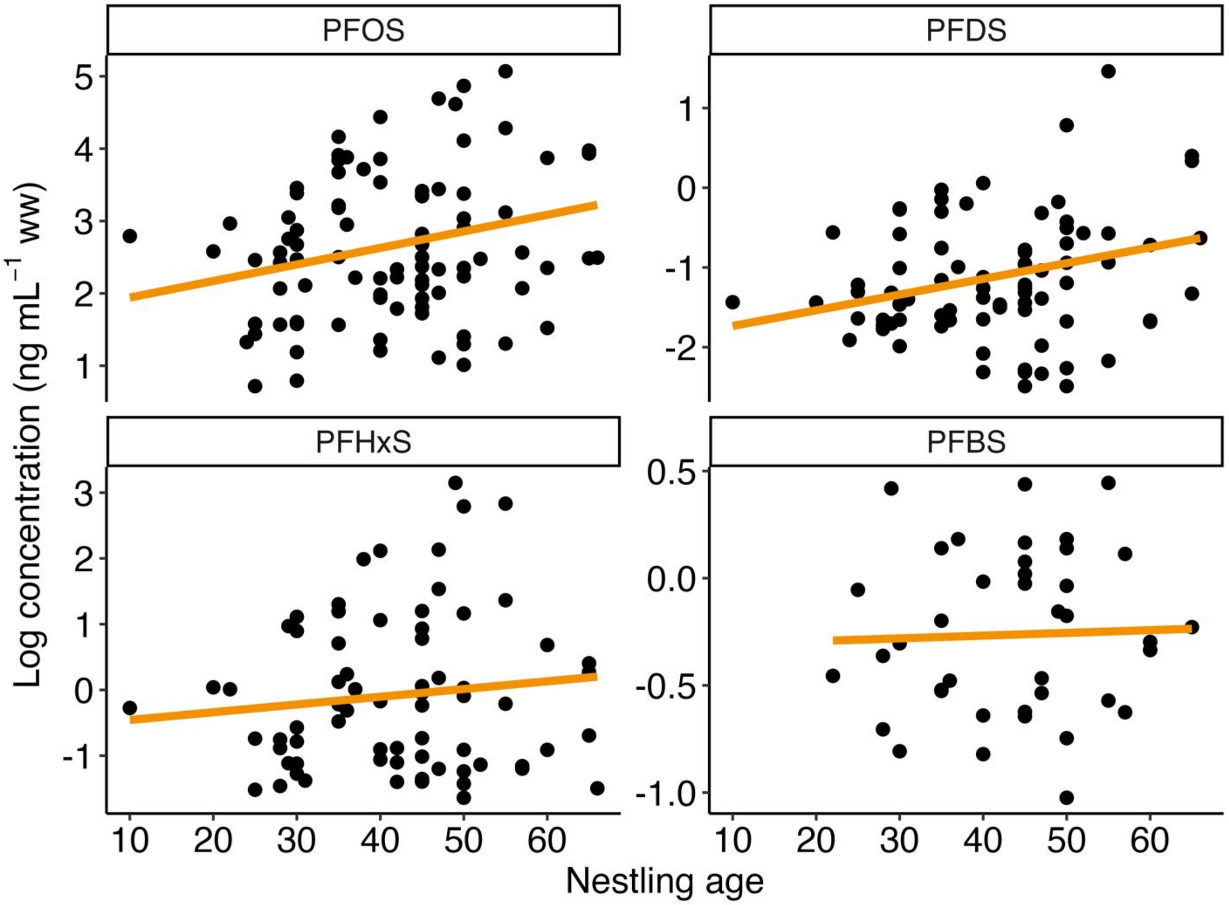

SI Figure 1. Relationship between PFSA concentrations and age in bald eagle (*Haliaeetus leucocephalus*) nestling plasma (*n* = 89) sampled between 2021 and 2023 across British Columbia (BC), Canada. Black dots represent individual data points. Orange trendline represents the linear mixed effects model between each PFSA concentration and nestling age as a fixed effect and nest site as a random effect.

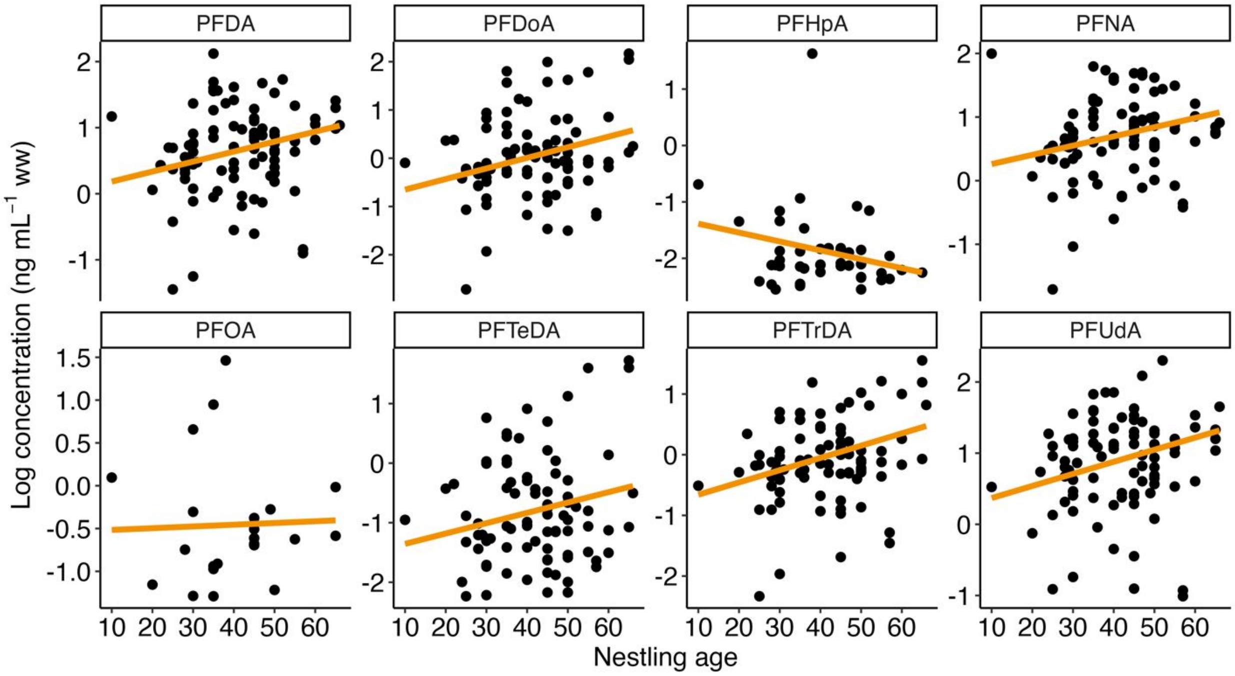

SI Figure 2. Relationship between PFCA concentrations and age in bald eagle (*Haliaeetus leucocephalus*) nestling plasma (*n* = 89) sampled between 2021 and 2023 across British Columbia (BC), Canada. Black dots represent individual data points. Orange trendline represents the linear mixed effects model between each PFCA concentration and nestling age as a fixed effect and nest site as a random effect.

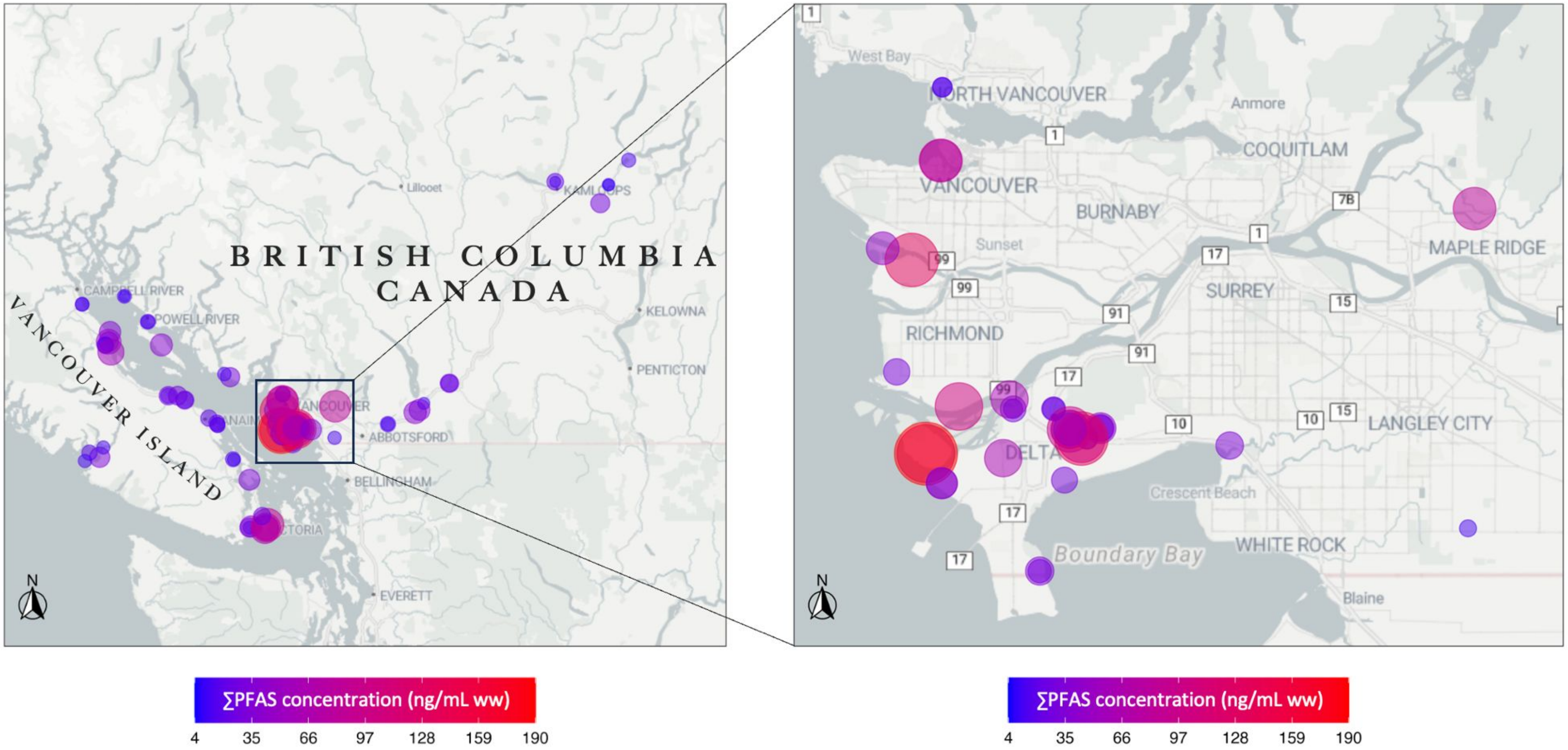

SI Figure 3. Geospatial plots visualizing levels of total perfluoroalkyl substance (∑PFAS) concentrations (ng/mL wet weight) in bald eagle (*Haliaeetus leucocephalus*) nestling plasma (*n* = 89) from British Columbia, Canada, 2021 to 2023.
